# Supplementary material for: The genome sequence of the leafhopper, Arthaldeus pascuellus (Fallén, 1826) (Hemiptera: Cicadellidae)
Source: Wellcome Open Res. 2026 Jan 6;11:17. [Version 1] doi: 10.12688/wellcomeopenres.25414.1 (PMC12859417; doi:10.12688/wellcomeopenres.25414.1)
Supplement: Peer review history

# Reviewer report: Reviewer response for version 1

**DOI:** 10.21956/wellcomeopenres.28000.r144896

**Published:** 2026-01-30

**Authors:** Qianquan Chen

**Affiliations:** 1 Guizhou Normal University, Guiyang, Guizhou, China

*Arthaldeus pascuellus *is a small deltocephaline leafhopper, which is widely distributed in the UK. *A. pascuellus* belongs to the representative of British Cicadellidae which is a diverse and ecologically important family of herbivorous Hemiptera. A chromosome-level genome sequence was decoded by using HiFi and Hi-C sequencing. Methods are presented clearly in the main text. The genome sequence can contribute to the research of *A. pascuellus*.

Are sufficient details of methods and materials provided to allow replication by others?

Yes

Is the rationale for creating the dataset(s) clearly described?

Yes

Are the datasets clearly presented in a useable and accessible format?

Yes

Are the protocols appropriate and is the work technically sound?

Yes

Reviewer Expertise:

Eco-genomics

I confirm that I have read this submission and believe that I have an appropriate level of expertise to confirm that it is of an acceptable scientific standard.

# Reviewer report: Reviewer response for version 1

**DOI:** 10.21956/wellcomeopenres.28000.r144217

**Published:** 2026-01-24

**Authors:** Sam T Mugford

**Affiliations:** 1 John Innes Centre, Norwich Research Park, Norwich, UK

Very clearly written report, I have only minor queries:

 The sample collection methods indicate a single individual was used for Hi-C, and the Hi-C methods describe an input of 20-50mg tissue which sounds a lot for a single leafhopper. Are they this big? Or were more than one used in HiC.

 The use of a space in the genome size "1 275.44 Mb" is confusing, I suggest a comma "1,275.44"

 Is *Arthaldeus *predominantly a phloem- or xylem-feeder? and is it a known vector of plant pathogens? (this might be useful to include in the introduction)

 Is there any insight into obligate/facultative endosymbionts present from the Blobtools contamination analysis?

Are sufficient details of methods and materials provided to allow replication by others?

Yes

Is the rationale for creating the dataset(s) clearly described?

Yes

Are the datasets clearly presented in a useable and accessible format?

Yes

Are the protocols appropriate and is the work technically sound?

Yes

Reviewer Expertise:

molecular plant-insect interactions

I confirm that I have read this submission and believe that I have an appropriate level of expertise to confirm that it is of an acceptable scientific standard.
